# Supplementary material for: An artificial visual neuron with multiplexed rate and time-to-first-spike coding
Source: Nat Commun. 2024 May 1;15:3689. doi: 10.1038/s41467-024-48103-9 (PMC11063071; doi:10.1038/s41467-024-48103-9)
Supplement: Supplementary file 1 — Supplementary Information [file 41467_2024_48103_MOESM1_ESM.pdf]

Supplementary Information

An artificial visual neuron with multiplexed rate and time-to-first-spike coding

Fanfan Li[1,2]†, Dingwei Li[2]†, Chuanqing Wang[3]†, Guolei Liu[2], Rui Wang[4], Huihui Ren[2], Yingjie Tang[2], Yan Wang[2], Yitong Chen[2], Kun Liang[2], Qi Huang[5], Mohamad Sawan[3,5,6], Min Qiu[2,5,6], Hong Wang[4]*, Bowen Zhu[2,5,6]*

†These authors contributed equally to this work.

1. School of Materials Science and Engineering, Zhejiang University, Hangzhou 310027, China.
2. Key Laboratory of 3D Micro/Nano Fabrication and Characterization of Zhejiang Province, School of Engineering, Westlake University, Hangzhou 310024, China.
3. CenBRAIN Neurotech, School of Engineering, Westlake University, Hangzhou 310024, China.
4. Key Laboratory of Wide Band Gap Semiconductor Technology, School of Microelectronics, Xidian University, Xi'an, 710071 China.
5. Westlake Institute for Optoelectronics, Westlake University, Hangzhou 311421, China.
6. Institute of Advanced Technology, Westlake Institute for Advanced Study, Hangzhou 310024, China.

*Corresponding authors: Email: zhubowen@westlake.edu.cn (B.Z.); hongwang@xidian.edu.cn (H.W.)

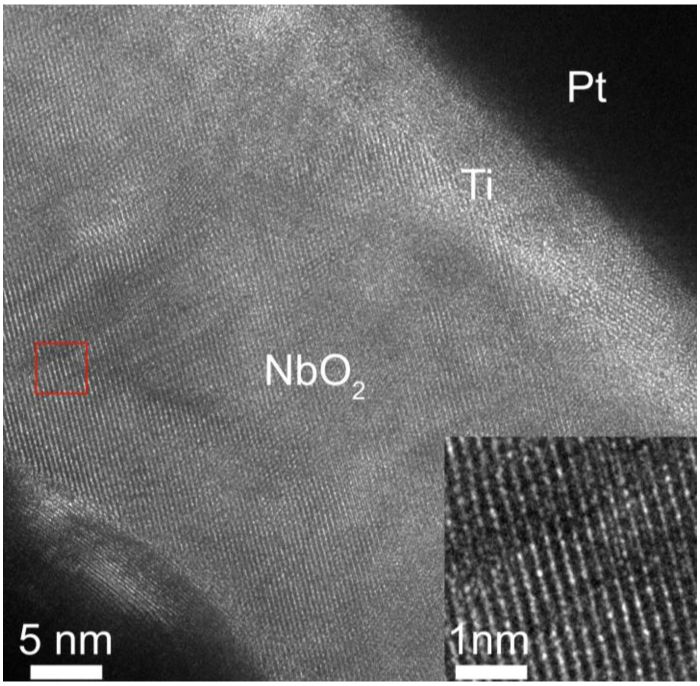

Supplementary Fig. 1. Transmission electron microscopy (TEM) image of the NbO_x Mott memristor. Inset shows high resolution TEM image corresponding to the red square area, showing locally crystallized region of NbO_2.

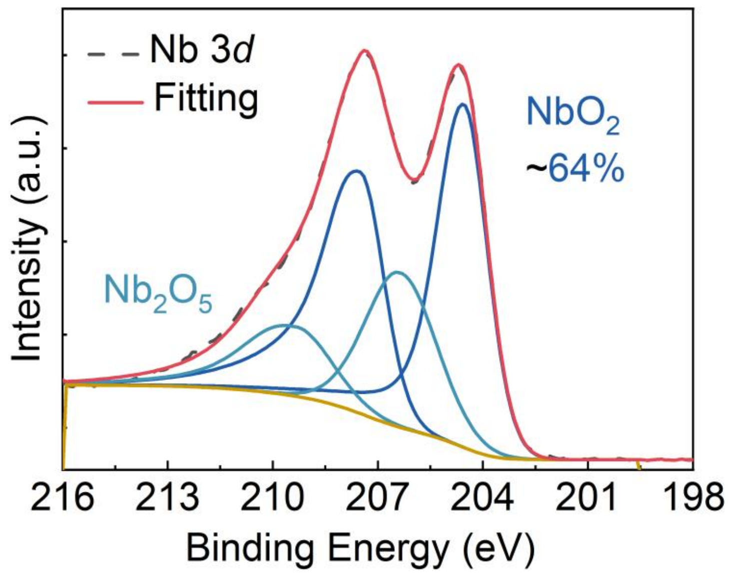

Supplementary Fig. 2. X-ray photoelectron spectroscopy (XPS) analysis of NbO_x films.

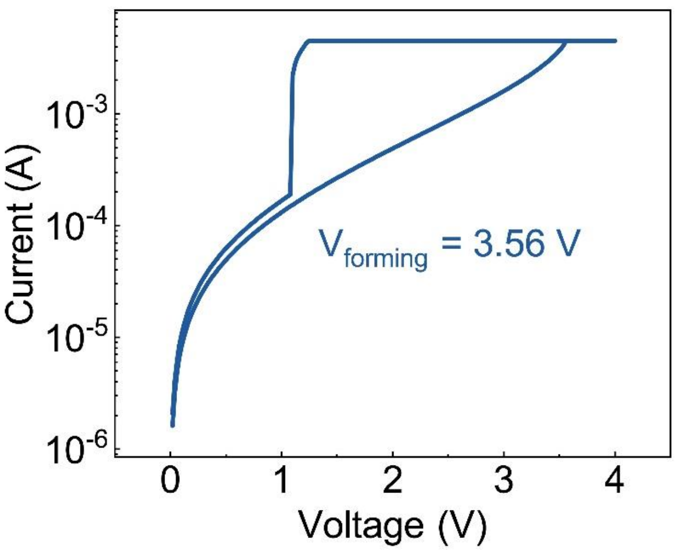

Supplementary Fig. 3. Electroforming operation of the NbO_x memristor. A forming voltage with a compliance current of 4 mA was applied to activate reproducible threshold switching behaviors.

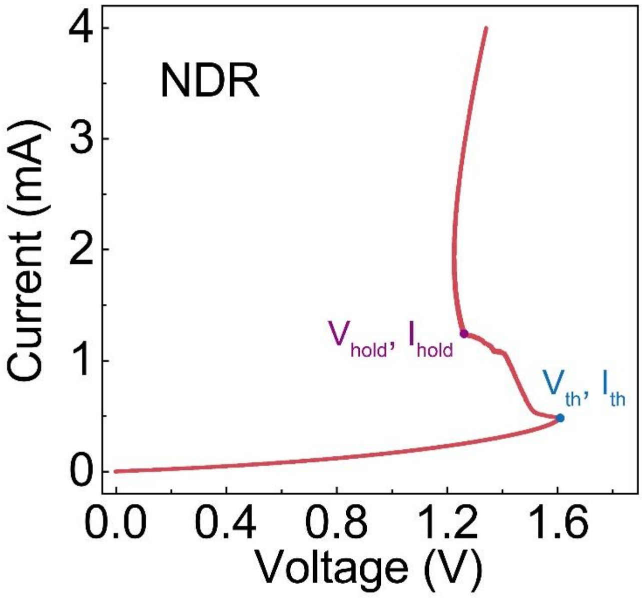

Supplementary Fig. 4. Current-programmed negative differential resistance (NDR) characteristics of the NbO_x memristor.

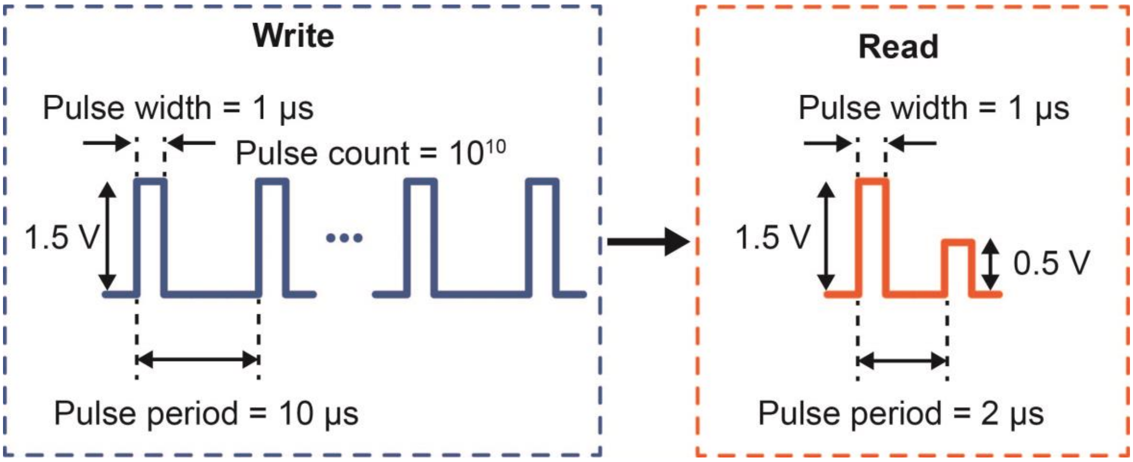

Supplementary Fig. 5. Pulse operation with the endurance characteristics of the NbO_x Mott memristors.

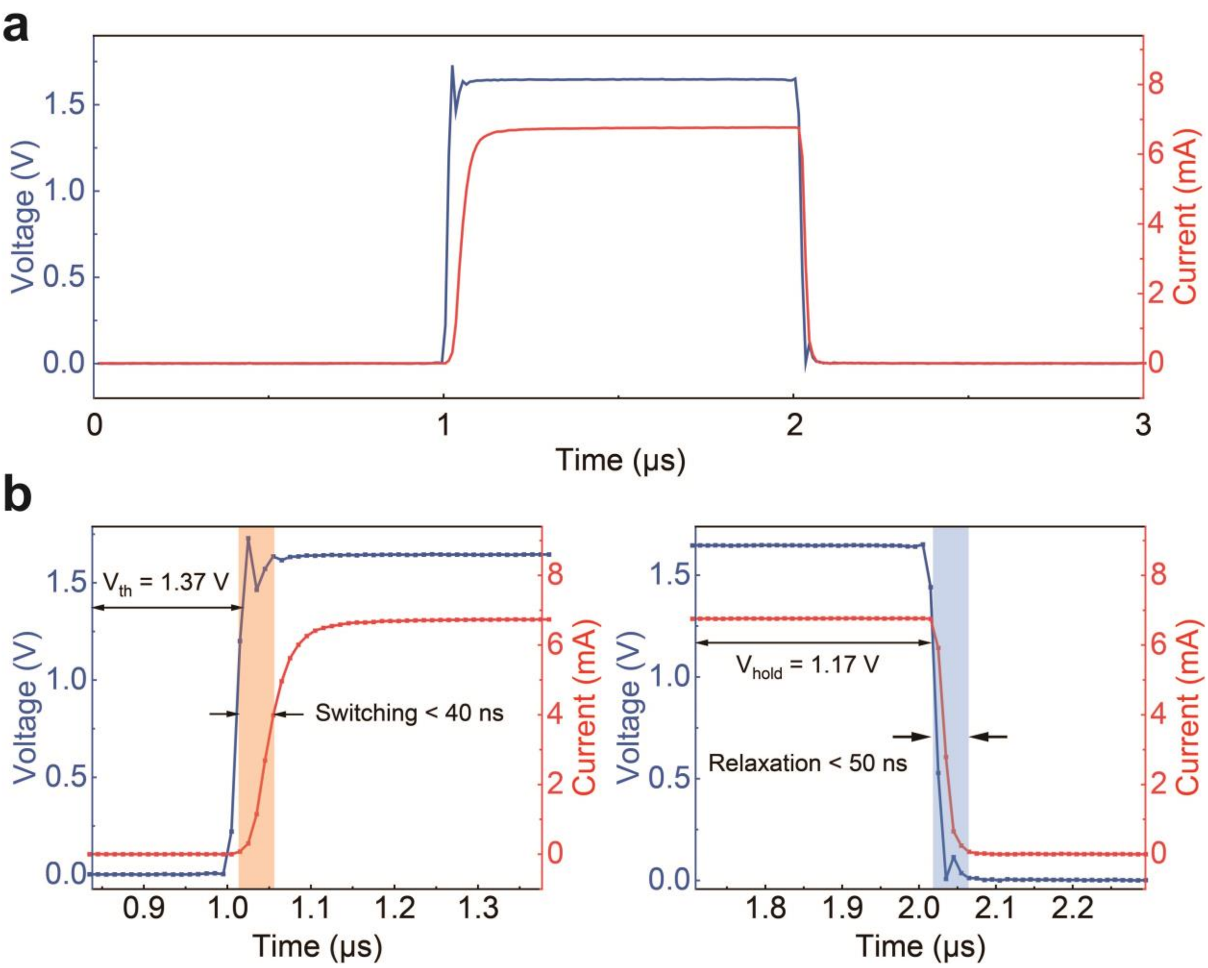

Supplementary Fig. 6. Transient switching response of the NbO_x memristor. a, The programmed voltage-pulse input (blue curve) and corresponding current response (red curve). **b,** The switching speed is < 40 ns from off-state to on-state (left) and is < 50 ns from on-state to off-state (right).

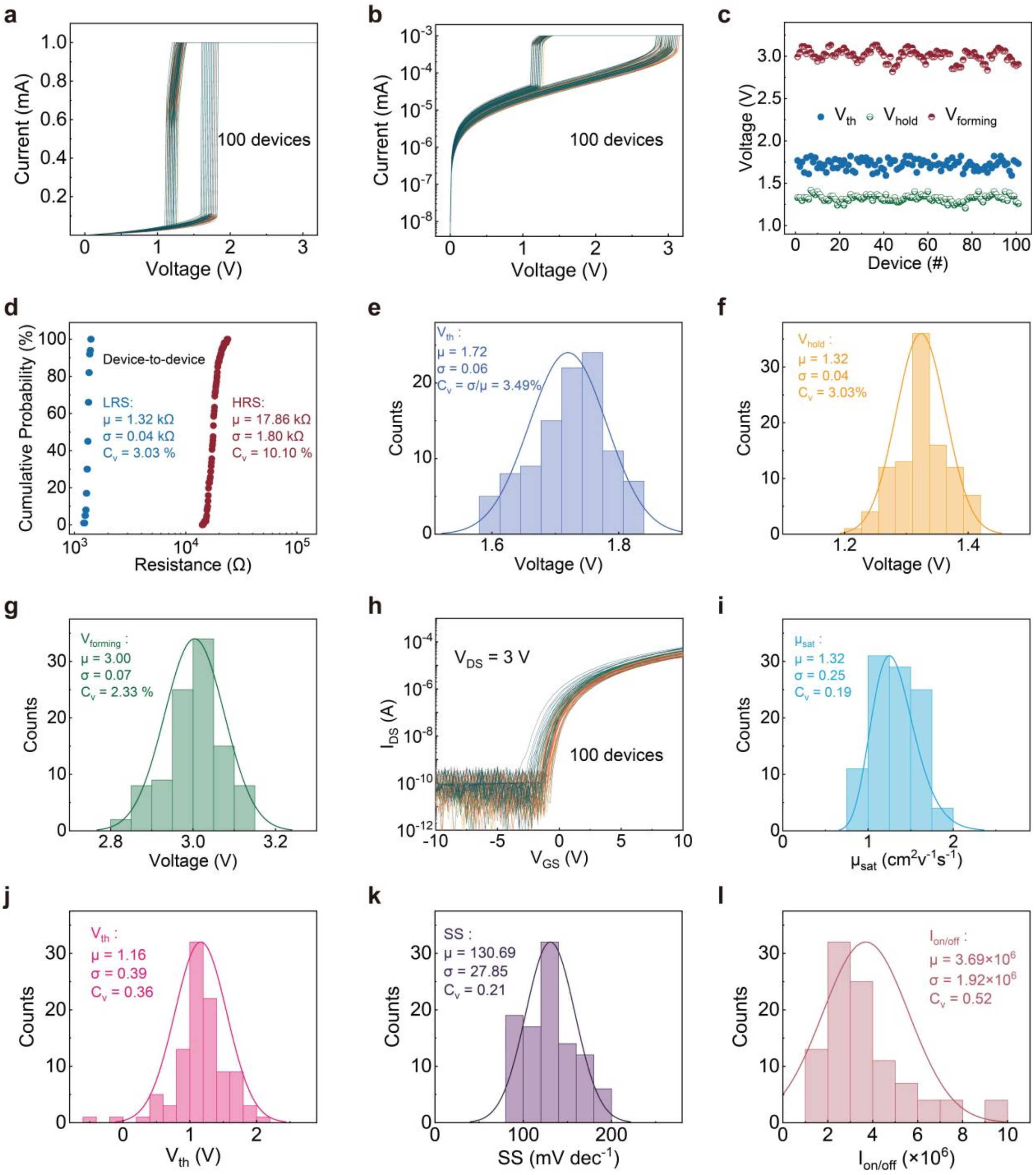

Supplementary Fig. 7. Device-to-device variation of 100 artificial visual neurons on 2-inch silicon wafer. a, Current-voltage (I-V) curves with threshold switching characteristics of 100 NbO_x Mott memristors with a working area of 1 μm × 1 μm. **b,** Electroforming operation of the 100 NbO_x memristor. **c,** The V_{th}, V_{hold} and $V_{forming}$ of 100 NbO_x memristors. **d,** Device-to-device variation of R_{LRS} and R_{HRS} for 100 devices. **e-g,** The statistical analysis of variability of V_{th} (**e**), V_{hold} (**f**) and $V_{forming}$ (**g**) in 100 NbO_x memristors. **h,** Transfer characteristics at a source-to-drain voltage (V_{DS}) of 3V and measured in the dark for 100 In_2O_3 phototransistors with channel lengths (L) of 10 μm and channel widths (W) of 400 μm. **i-l,** Device-to-device variation is represented using histograms of saturation mobility values (μ_{sat}) (**i**), threshold voltage (V_{th}) (**j**), subthreshold slopes (SS) (**k**) and current on/off ratios ($I_{on/off}$) (**l**).

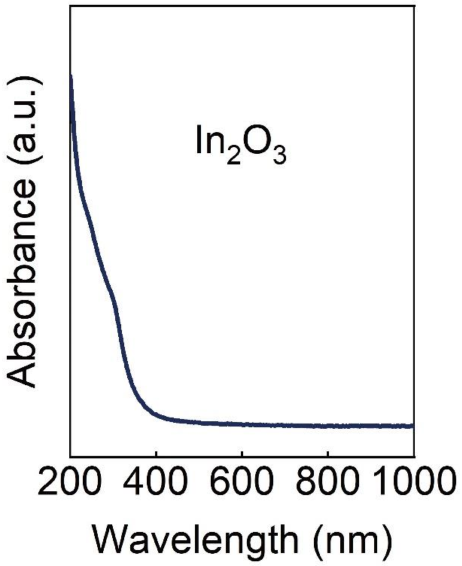

Supplementary Fig. 8. Ultraviolet-visible absorbance spectrum of In_2O_3 film.

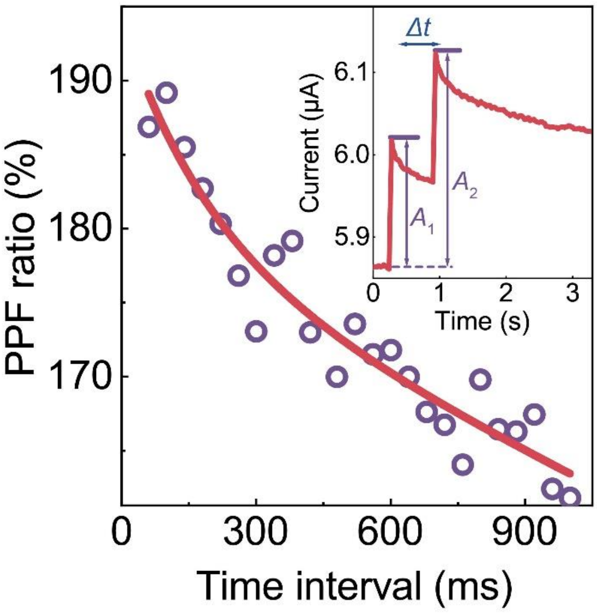

Supplementary Fig. 9. Paired pulse facilitation (PPF) ratio as a function of the optical pulse interval. The light intensity is 1.71 mW/cm^2 and the pulse width is 5 ms. PPF index is defined as $(A_2-A_1)/A_1\times100\%$, where the red line represents fitting results using the exponential decay function.

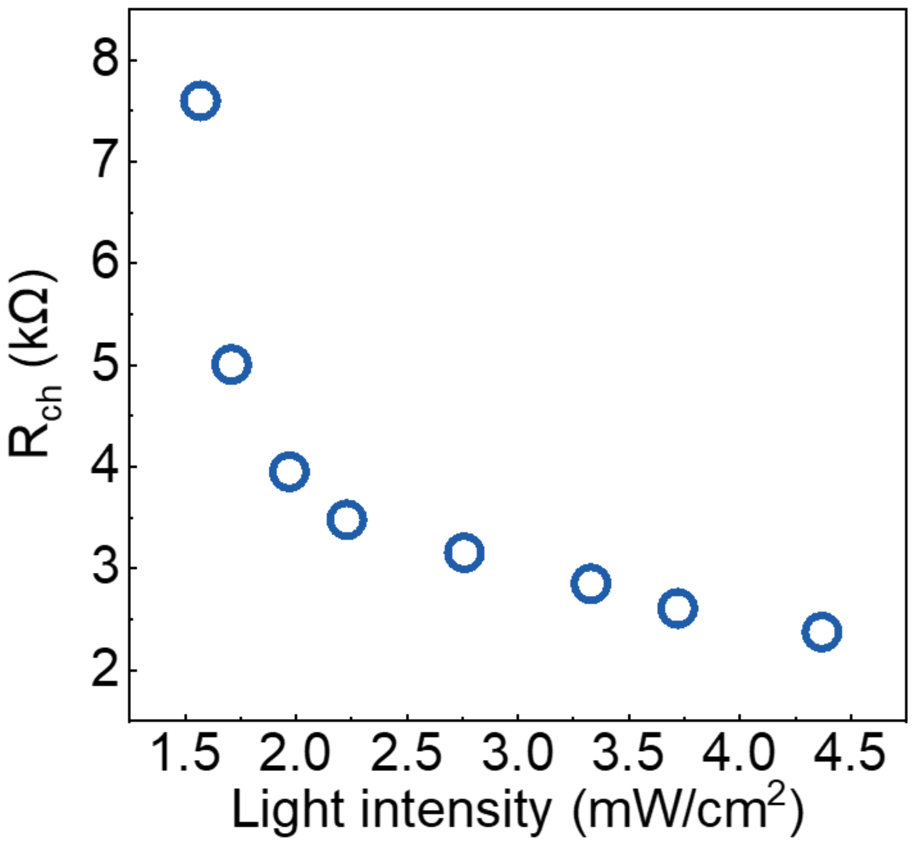

Supplementary Fig. 10. The change in R_{ch} of In_2O_3 phototransistor as a function of light power density (from 1.57 to 4.37 mW/cm^2, λ = 365 nm). R_{ch} measured at a DC bias of V_{DS} = 3 V and V_{GS} = 5 V.

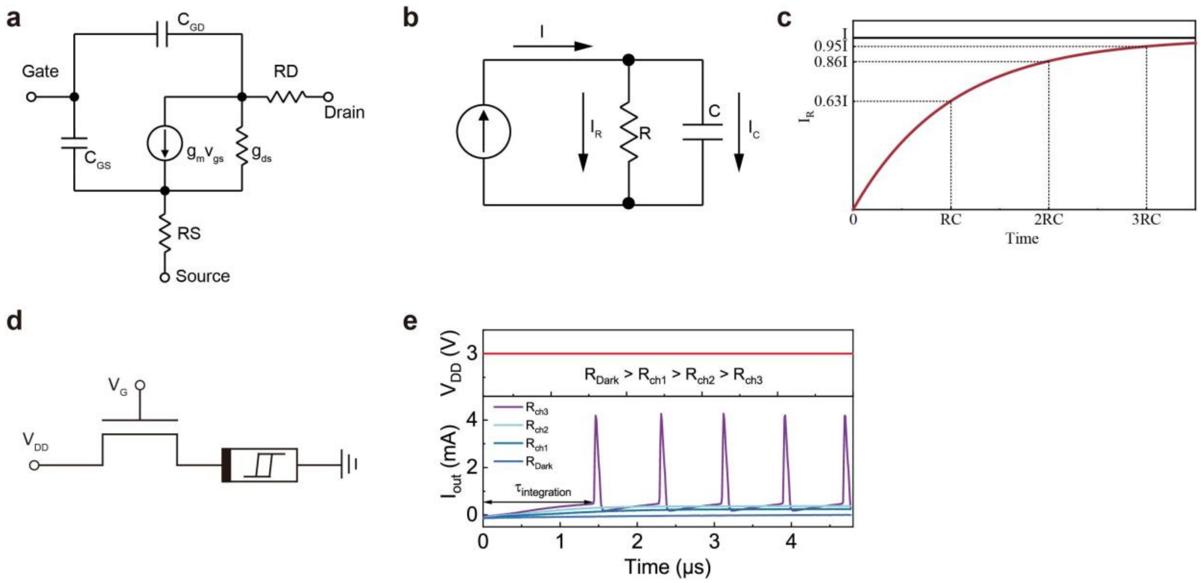

Supplementary Fig. 11. Response of the the artificial visual neuron. a, A small-signal model of an oxide TFT. **b,** The equivalent circuit of the TFT comprises a resistor and a capacitor connected in parallel, without considering contact resistance, and is then linked to a constant current source. **c,** The change in current across the equivalent circuit resistance. **d,** Schematic circuit of 1T1R synapses and neurons. **e,** The optical pulse and the current waveform were regarded as the input and output signals, respectively. An electric pulse (V_{DD}: 3 V, 20 μs) along with a V_G electric pulse (3 V, 20 μs) was applied to read the spike behaviors. As light pulse number increases, R_{ch} decreases (365 nm, 1.71 mW/cm^2, 5 ms, 0, 20, 40, and 100 cycles correspond to R_{Dark}, R_{ch1}, R_{ch2}, and R_{ch3}, respectively), resulting in a smaller time constant ($\tau_{integration}$). This, in turn, leads to a faster current reaching the firing threshold, resulting in the quicker arrival of the first spike. Additionally, influenced by rapid threshold switching, the charging and discharging during periodic spiking are incomplete, leading to faster charging and discharging times compared to the first spike.

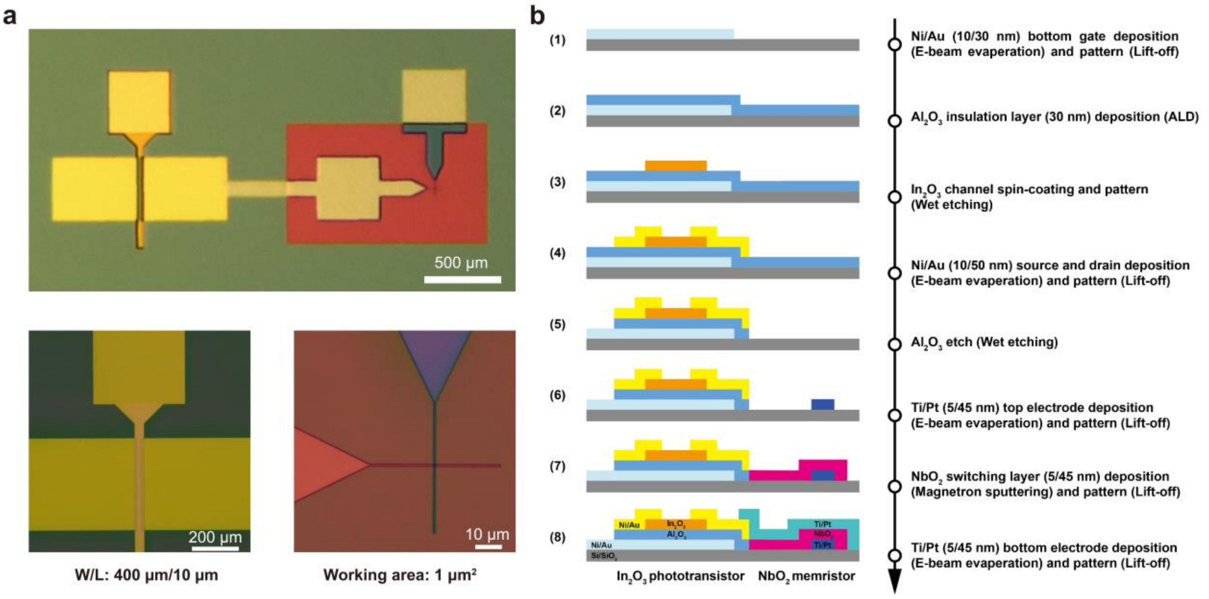

Supplementary Fig. 12. Monolithically integrated device and fabrication process. a, Optical image of compactly integrated In_2O_3 optoelectronic synaptic transistors and NbO_x Mott neurons (1T1R). **b,** The flow chart of 1T1R fabrication process.

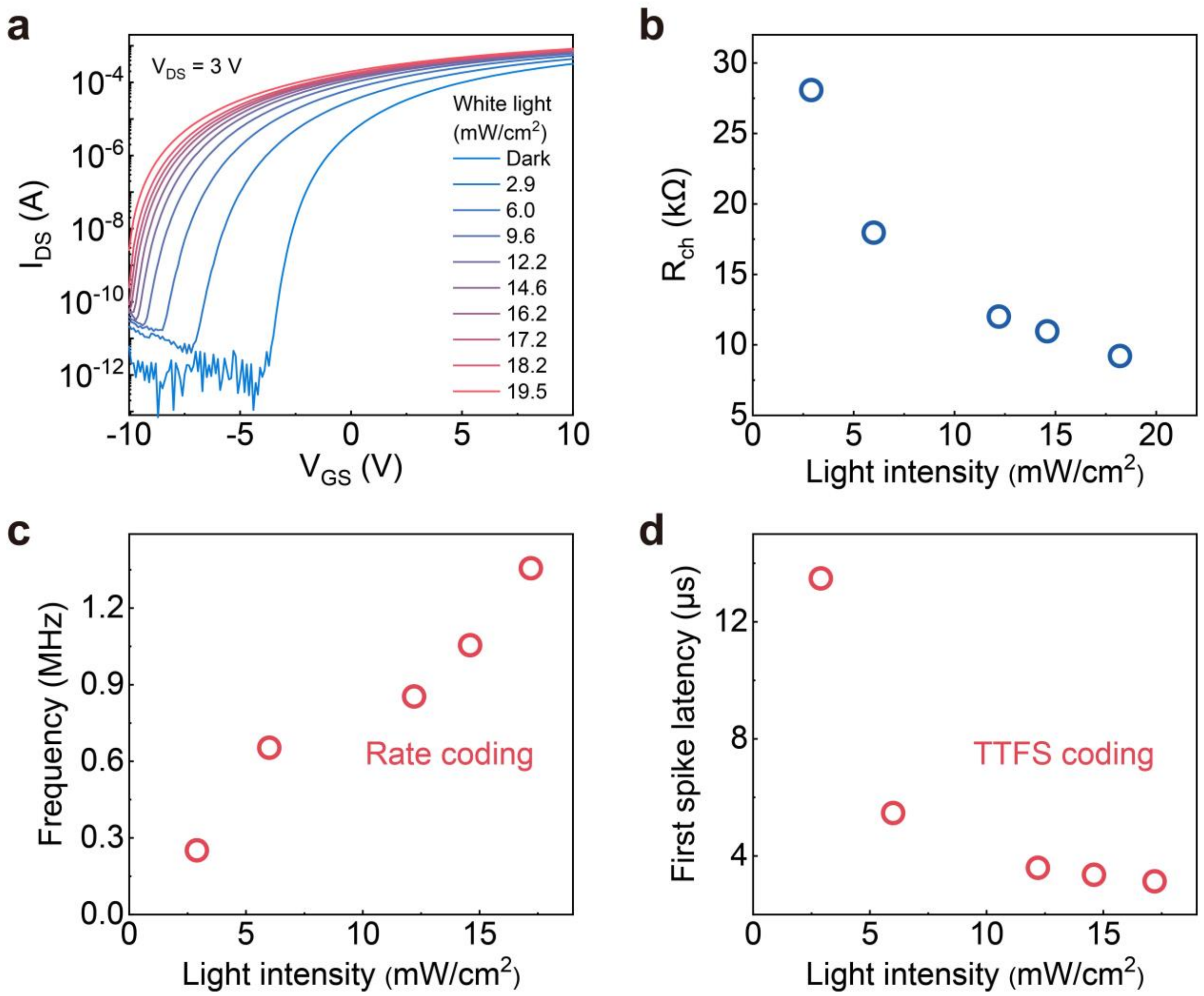

Supplementary Fig. 13. Rate-temporal fusion photo-encoding under white light. a, Transfer curves of the In_2O_3 phototransistor as a function of light power density (from 2.9 to 19.5 mW/cm^2, white light). I_{DS} versus V_{GS} measured at a drain bias of V_{DS} = 3 V. **b,** The change in R_{ch} of In_2O_3 phototransistor as a function of light power density (V_{DS} = 3 V and V_{GS} = 3 V). **c,d,** The effect of the light intensity on the (**c**) spike frequency and (**d**) first spike latency (V_{DD} = 3.7 V, V_G = 3 V, 20 μs width and 1s duration of illumination).

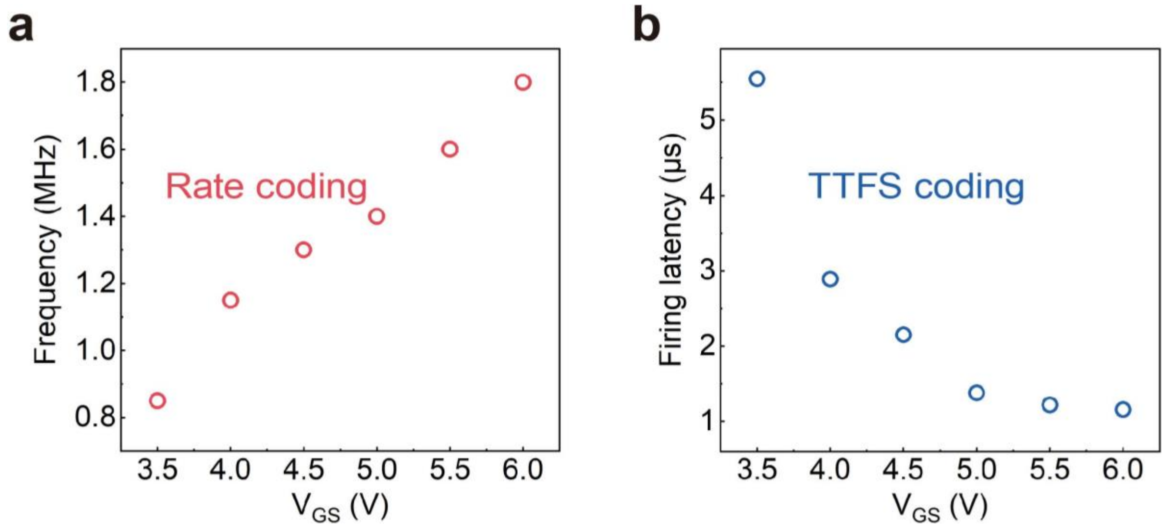

Supplementary Fig. 14. The effect of gate voltage on spike frequency and first spike latency. a, The effect of gate voltage (V_{GS}) on spike frequency. **b,** The effect of V_{GS} on first spike latency. An electric pulse V_{DD} (3 V, 20 μs) was applied to read the spike behaviour in response to 100 optical pulses (λ = 365 nm, 2.76 mW/cm^2, 5 ms, 100 Hz).

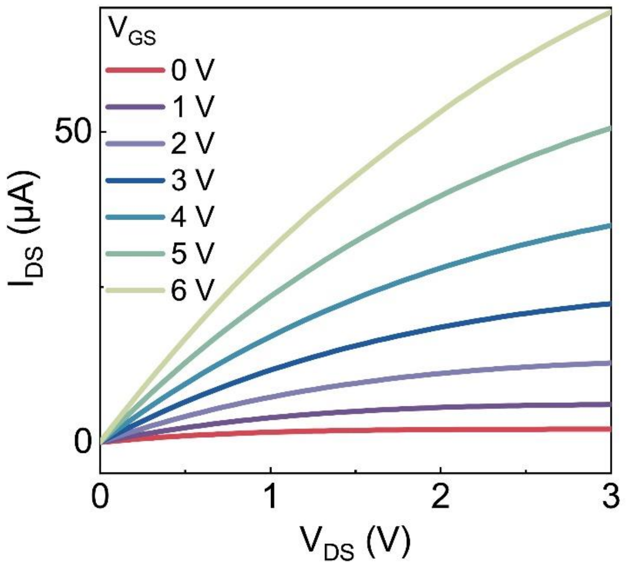

Supplementary Fig. 15. Output characteristics of optoelectronic synaptic transistor with V_G changed from 0 to 6 V in steps of 1 V.

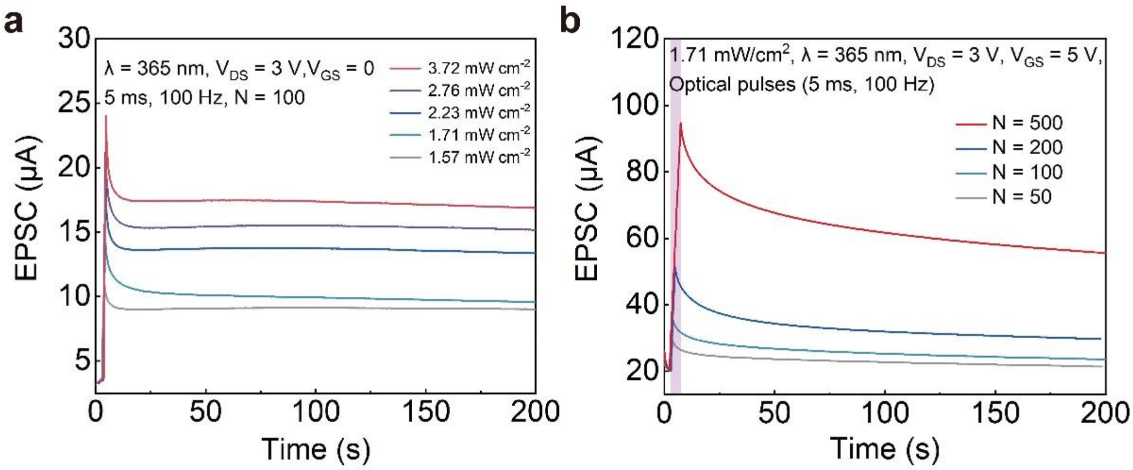

Supplementary Fig. 16. Synaptic plasticity under optical modulation. a, Excitatory postsynaptic current (EPSC) of the optoelectronic synaptic transistor triggered by various ultraviolet (UV) illumination intensities. **b,** EPSC of the optoelectronic synaptic transistor triggered by various UV light pulse numbers.

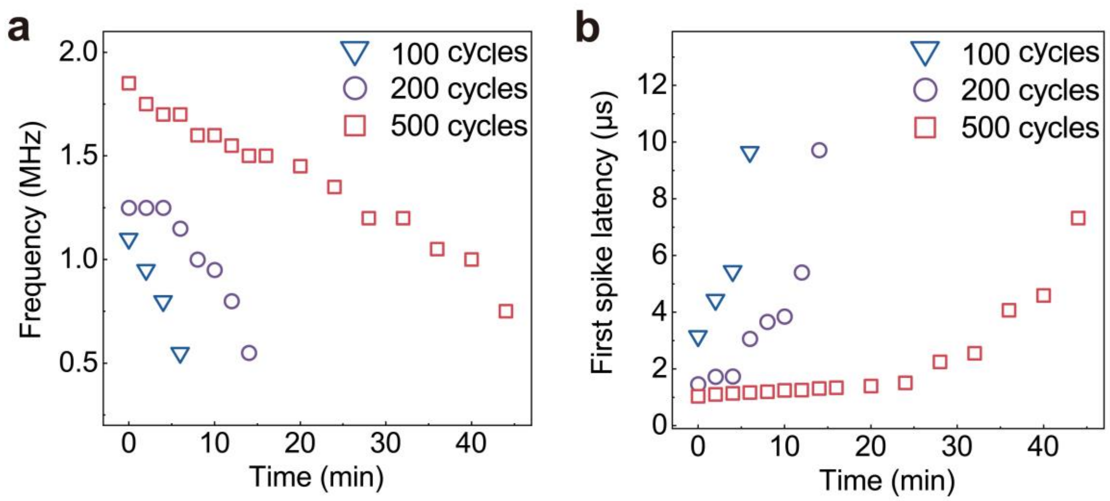

Supplementary Fig. 17. The rate-temporal fusion (RTF) coding performance over time in dark after UV light illumination with different pulse numbers. a, Change in spike frequency. **b,** Change in first spike latency.

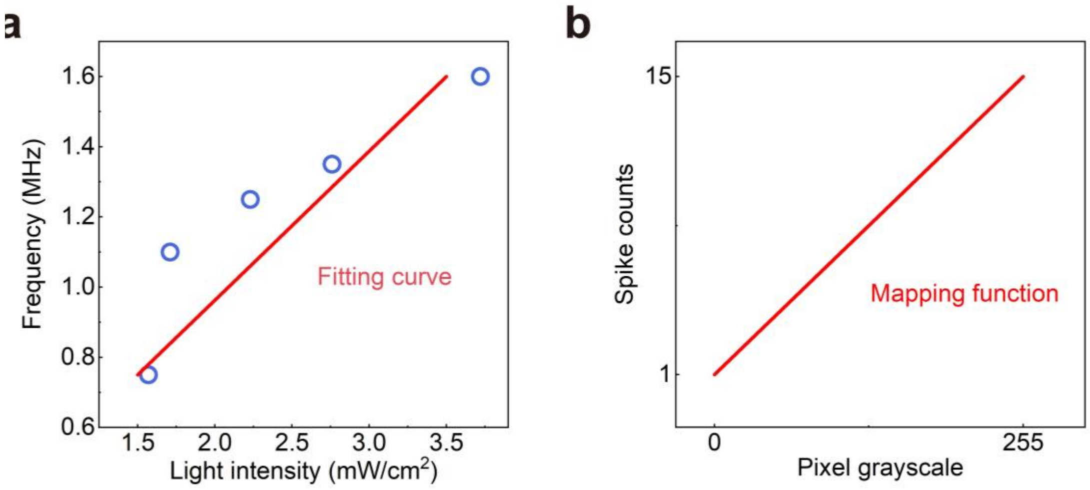

Supplementary Fig. 18. Spike frequency as a linear function of the light intensity. a, The artificial visual spiking neuron exhibits a tunable spike frequency response characteristic under varying light intensities. **b,** Mapping function depicting the relationship between spike counts and varying pixel grayscale. The red line indicates the fitted curve parameters, which was utilized in the mapping algorithm.

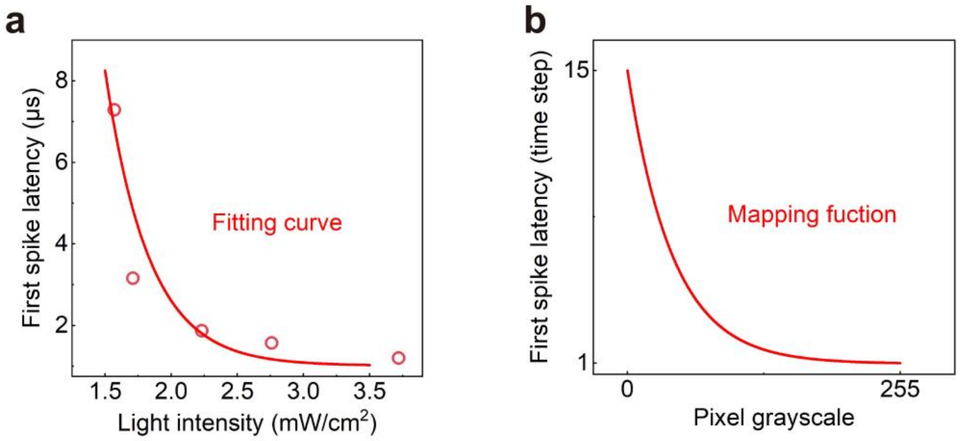

Supplementary Fig. 19. First spike latency time as an exponential function of the light intensity. a, The artificial visual spiking neuron exhibits a tunable first spike latency response characteristic under varying light intensities. **b,** Mapping function depicting the relationship between first spike latency and varying pixel grayscale. The red line indicates the fitted exponential decay function, which was utilized in the mapping algorithm.

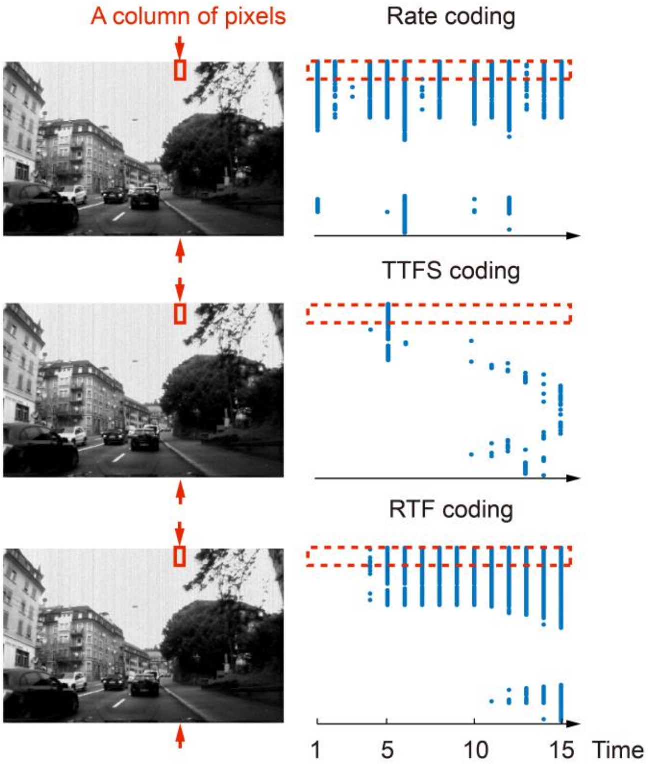

Supplementary Fig. 20. Spike raster of a self-driving recording image for rate coding, TTFS coding, and RTF coding in 15 time-steps. Spike raster of a self-driving recording image for rate coding, TTFS coding, and RTF coding in 15 time-steps. Spike trains of different coding methods for image locations along the column are marked by arrows. The spike sequence depicted within the red dashed box is derived from the pixels enclosed by the red box. In rate coding, there exists a linear relationship between the pixel grayscale value and the frequency of the resulting spike train. Conversely, in TTFS coding, the pixel grayscale is connected to the first spike latency via an exponential decay function. The generated spike train in RTF coding incorporates these two fitting relationships.

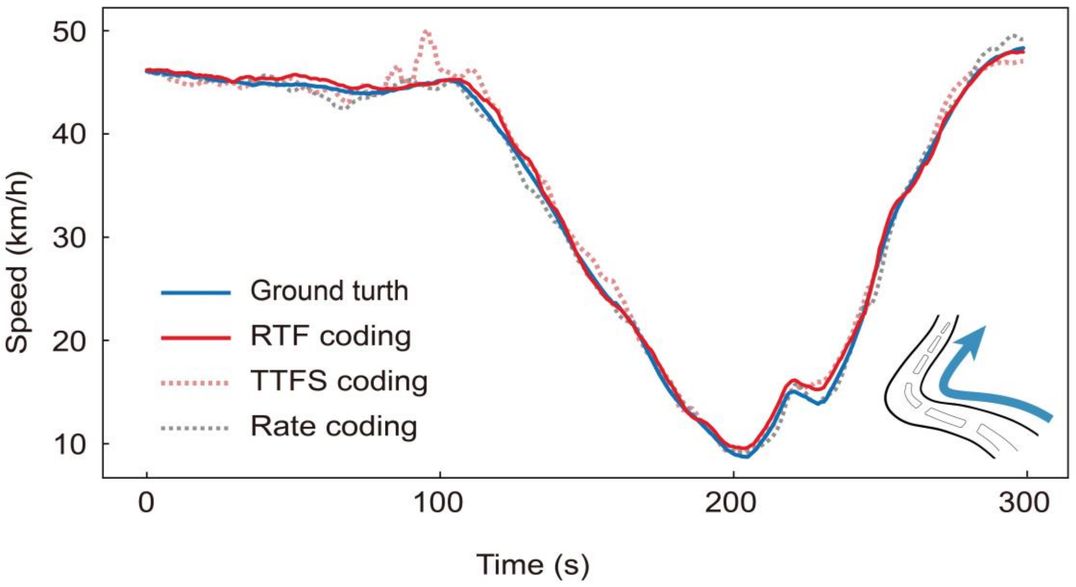

Supplementary Fig. 21. The comparison of speed prediction results of rate coding, TTFS coding, and RTF coding in complex road corners.

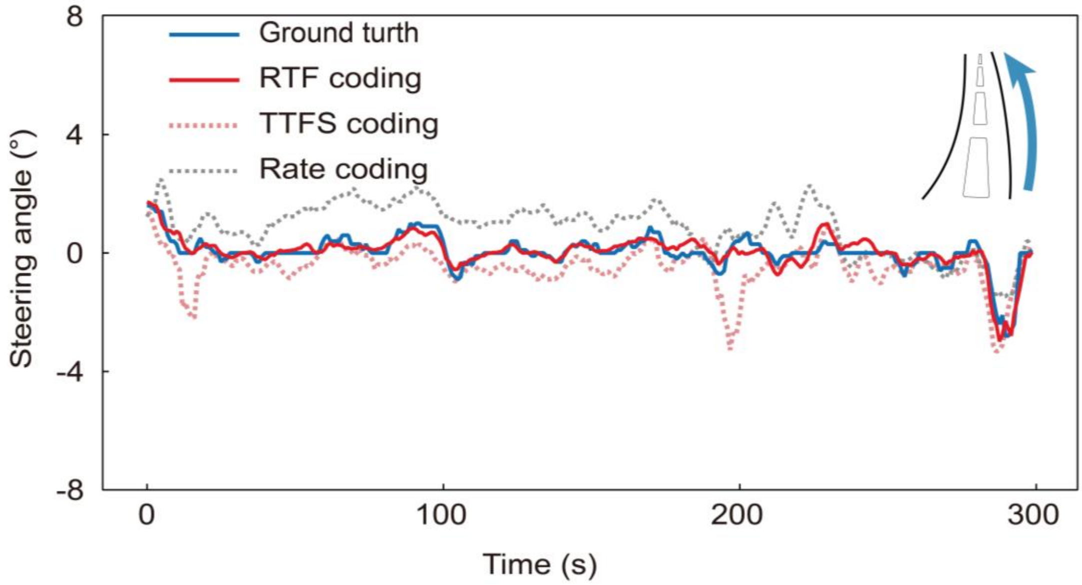

Supplementary Fig. 22. The comparison of steering angle prediction results of rate coding, TTFS coding and RTF coding in smooth road corner.

Supplementary Table 1. Comparison of different artificial spiking visual neurons.

Materials	Mechanism	Components	Switching/Relaxation speed	Endurance	Ref.
Two-dimensional b-AsP/$MoTe_2$	Optoelectronic	1T	NA	NA	1
Metal oxide	Filament-based	1D1T	40/55 ns	>500	2
Silicon	Electronic	CMOS circuit	NA	NA	3
Silicon	Optoelectronic	1T	NA	NA	4
Metal oxide	Filament-based	$2R1C1R_M$	10/40 ns	>1000	5
Metal oxide	Filament-based	$1R1C2R_M$	16/21 ns	>50	6
Monolayer MoS_2	Optoelectronic	21T	NA	NA	7
**Metal oxide**	**Mott transition**	**$1T1R_M$**	**20/38 ns**	**$>10^{10}$**	**This work**

Abbreviations: Transistor (T), diode (D), resistor (R), capacitor (C), memristor (R_M).

Supplementary Table 2. Comparison of characteristics of various artificial spiking visual neurons.

Neuron coding			Excitatory and inhibitory	Spike integration	Synaptic plasticity	Spiking frequency (Hz)	Spiking time resolution (s)	Energy/spike	Ref.
Rate	TTFS	TRF							
Yes	No	No	No	No	No	$0\text{-}10^5$	No	NA	1
Yes	No	No	No	Yes	No	0.1-1200	No	~40 pJ	2
No	Yes	No	Yes	Yes	Yes	9.6	~0.23	~93 mJ	3
Yes	No	No	Yes	Yes	No	35-60	No	~6.8 nJ	4
Yes	No	No	No	Yes	No	1-200	No	~2.5 nJ	5
Yes	No	No	No	Yes	Yes	0-160	No	NA	6
No	Yes	No	No	Yes	No	No	0.7	0.1-1 μJ	7
**Yes**	**Yes**	**Yes**	**Yes**	**Yes**	**Yes**	**$(0.35\text{-}1.85) \times 10^6$**	**1.04×10^{-6}**	**1.06 nJ**	**This work**

Supplementary Note 1. Rate coding technique.

The artificial visual spiking neuron composed of an In_2O_3 synaptic phototransistor and a NbO_x memristor exhibits a tunable spike frequency response characteristic under varying light intensities. The relationship between the spike frequency and light intensity can be modeled through a linear function, based on data recorded from our fabricated device, as depicted in Supplementary Fig. 18a. The fitting function is outlined as follows:

$$y = 0.1125 + 0.425\ x$$

where y and x represent spike frequency and light intensity, respectively.

Then, utilize the same linear fitting function to obtain the spike counts on different pixel grayscale, as shown in Supplementary Fig. 18b. The pixel grayscale also ranges from 0 to 255, and the spike counts is in the range of 1 to 15. The obtained spike frequency is a float number, which needs to conduct a floor operation to get an integer number. Finally, generate spike trains according to this spike frequency where the spike number and spike time follow a Poisson distribution.

To make it clear, here are the specific rate coding steps list below:

Algorithm 1: Rate coding

1: Fitting the relationship between spike frequency (MHz) and light intensity where the data is recorded from the fabricated device;

2: Based on the same fitting function, calculate the spike frequency (time step) under different pixel grayscale of the original image;

3: Conduct a floor operation to obtain spike frequency (integer number);

4: Generate a spike train according to this spike frequency where the spike frequency and spike time follow a Poisson distribution.

Supplementary Note 2. TTFS coding technique

The artificial visual spiking neuron exhibits a first-spike latency characteristic under varying light intensities. The relationship between the first spike latency and light intensity can be well fitted by an exponential decay function, as shown in Supplementary Fig. 19a. The fitting function is listed as follows:

$$T_{latency} = 1.01 + 675 \exp(-3.02\ x)$$

where $T_{latency}$ represents the time-to-first-spike, and x represents the light intensity.

Then, we utilize exponential decay function to obtain the first spike latency on different pixel grayscale, as shown in Supplementary Fig. 19a. The pixel grayscale ranges from 0 to 255,

and the first spike latency is in the range of 15 to 1. The obtain first spike latency is float number, which need to conduct floor operation to get an integer number. Finally, generate spike trains where the time of this only spike is the first spike latency (integer number).

To make it easier to understand, here are the specific TTFS coding steps below:

Algorithm 2: TTFS coding

1: Fitting the relationship between first spike latency (μs) and light intensity where the data is recorded from the fabricated device;

2: Based on the same fitting function, calculate the first spike latency (time step) under different pixel grayscale of the original image;

3: Conduct a floor operation to obtain the first spike latency (integer number);

4: Generate a spike train where the time of this only spike is the first spike latency (integer number).

Supplementary Note 3. RTF coding technique

The RTF coding incorporates the above two features, where the spike frequency (F_{Rate}) and first spike latency are obtained using the same fitting function with rate and TTFS coding techniques, respectively. Then, it is necessary to calculate the number of time steps (F_{rest}) remaining after first spike in TTFS coding. If the F_{rest} is larger than F_{Rate}, the $F_{Final} = F_{Rate}$. Otherwise, $F_{Final} = F_{rest}$. First, ensure the location of the first spike using the result of TTFS coding. The rest of the spike train depends on the F_{Final}. If $F_{Final} = F_{Rate}$, the rest of spike train is generated according to the spike frequency from rate coding. If $F_{Final} = F_{rest}$, then every position after the first spike will have one spike.

Algorithm 3: RTF coding

1: Obtaining the first spike latency (time step) and spike frequency with the same function of TTFS and rate coding;

2: Calculating the number of time steps (F_{rest}) after first spike in TTFS coding;

3: Comparing the F_{rest} and F_{Rate} to obtain the final frequency F_{Final};

4: If $F_{Final} = F_{Rate}$, the rest of spike train is generated according to the spike frequency from rate coding;

5: If $F_{Final} = F_{rest}$, the all position after first spike has one spike.

Finally, utilize one original image to demonstrate all these three coding techniques, as shown in Supplementary Fig. 18. The pixel grayscale has a linear relationship with the

frequency of the generated spike train in rate coding. The pixel grayscale is related to the first spike latency in TTFS coding through an exponential decay function. The generated spike train in RTF coding incorporates these two fitting relationships.

Supplementary References

1 Wang, F. *et al.* A two-dimensional mid-infrared optoelectronic retina enabling simultaneous perception and encoding. *Nat. Commun.* **14**, 1938 (2023).

2 Wang, X. *et al.* Vertically integrated spiking cone photoreceptor arrays for color perception. *Nat. Commun.* **14**, 3444 (2023).

3 Kim, S. *et al*. Artificial stimulus-response system capable of conscious response. *Sci. Adv*. **7**, eabe3996 (2021).

4. Han J. K., et al. Bioinspired photoresponsive single transistor neuron for a neuromorphic visual system. *Nano. Lett.* **20**, 8781-8788 (2020).

5. Chen C., et al. A photoelectric spiking neuron for visual depth perception. *Adv. Mater.* **34**, e2201895 (2022).

6. Pei Y., et al. Artificial visual perception nervous system based on low-dimensional material photoelectric memristors. *ACS Nano* **15**, 17319-17326 (2021).

7. Subbulakshmi Radhakrishnan S., et al. A sparse and spike-timing-based adaptive photoencoder for augmenting machine vision for spiking neural networks. *Adv. Mater.* **34**, e2202535 (2022).
